# Acceptability of HPV Vaccination for Daughters: A University Hospital-Wide Questionnaire Survey

**DOI:** 10.3390/vaccines14030218

**Published:** 2026-02-27

**Authors:** Midori Yamaguchi, Akiko Sukegawa, Kenji Ohshige, Yukio Suzuki, Atsuko Furuno, Etsuko Miyagi, Taichi Mizushima

**Affiliations:** 1 Department of Obstetrics and Gynecology, Yokohama City University School of Medicine, Yokohama 236-0004, Japan; yamaguchi.mid.oo@yokohama-cu.ac.jp (M.Y.); aki69kin@yokohama-cu.ac.jp (A.S.); yetii@yokohama-cu.ac.jp (Y.S.); emiyagi@yokohama-cu.ac.jp (E.M.); 2 Center for Health Service Sciences, Yokohama National University, Yokohama 240-8501, Japan; kenoh@ynu.ac.jp; 3 Department of Gynecology, Kanagawa Cancer Center, Yokohama 241-8515, Japan; 4 Department of Obstetrics and Gynecology, Yokohama Minami Kyosai Hospital, Yokohama 236-0037, Japan; furuno.ats.us@yokohama-cu.ac.jp

**Keywords:** HPV vaccine, vaccine acceptability, medical professionals, non-medical staff, hypothetical scenarios, contingent valuation method, questionnaire survey, Japan

## Abstract

Background/Objectives: Japan has experienced a marked decline in human papillomavirus (HPV) vaccination coverage, reaching less than 1%, after the government suspended its proactive recommendation in 2013, following media reports of symptoms alleged to be adverse events caused by the vaccine. Although the recommendation was reinstated in 2022 after comprehensive safety reviews, vaccination rates have remained modest. We aimed to assess HPV vaccine acceptability and identify factors associated with acceptance among staff at a university hospital. Methods: We administered a web-based questionnaire in February 2024 to 2761 hospital employees, assessing demographic and professional characteristics, HPV-related knowledge, awareness about vaccine effectiveness, adverse events, and catch-up programs, as well as acceptability across four hypothetical scenarios reflecting publicly funded and self-funded vaccination programs. Logistic regression analyses were conducted to identify factors associated with acceptability. Results: Among 1132 respondents (response rate 41.0%), acceptability exceeded 75% in the publicly funded scenarios but was approximately 45% in the self-funded scenarios. In multivariable analyses of the publicly funded scenarios, younger age, being a medical professional, greater HPV vaccine knowledge levels, and awareness about HPV vaccine effectiveness or catch-up vaccination were positively associated with acceptability; awareness about adverse events showed negative associations. In the self-funded scenarios, women were less likely to accept vaccination, but greater knowledge levels and awareness of catch-up vaccination remained positively associated with acceptability. Conclusions: These findings suggest that strategies tailored to specific population characteristics are important for improving HPV vaccine acceptability.

## 1. Introduction

Cervical cancer is primarily caused by persistent infection with human papillomavirus (HPV) [1]. The prevention of HPV infection is a critical strategy not only for preventing cervical cancer but also for other HPV-related malignancies [2]. In August 2020, the World Health Organization (WHO) announced a global strategy for the elimination of cervical cancer, built upon three pillars: (1) HPV vaccination, (2) cancer screening, and (3) treatment and care. The target for HPV vaccination is that 90% of girls should receive the recommended doses by the age of 15 years [3]. HPV vaccination programs were introduced around 2007 in several countries, including those in Western Europe and Australia, and had been adopted in 147 countries by 2024. However, global vaccination coverage for girls under the age of 15 years remains only 31%, far below the WHO target [4]. Japan has particularly low coverage, with rates dropping below 1% at one point, substantially lagging behind most other developed countries [5]. Although Japan’s incidence and mortality rates for cervical cancer are moderate by global standards, they remain elevated compared with North American and Western European countries and with Australia. Moreover, unlike in these regions, both rates in Japan continue to show an upward trend [6,7].

Japan began publicly funded HPV vaccination in 2010, which was incorporated into the national immunization program in 2013. However, following reports of various symptoms in which a causal relationship with the vaccine could not definitively be ruled out, the government suspended active recommendation for HPV vaccines in June 2013 [5]. Owing to this history, the HPV vaccination rate in Japan declined sharply from approximately 80% for those born in fiscal year (FY) 1997 to less than 1% for girls born after FY 2002 [5]. In November 2021, the government concluded that the benefits of the vaccine outweigh the potential risks and resumed its recommendation. Proactive recommendation for HPV vaccination restarted in April 2022, along with initiation of a 3-year catch-up program designed to restore vaccination coverage and protect cohorts left unvaccinated during the period of suspension. In April 2023, the 9-valent vaccine was added to the publicly funded program. Despite these policy changes, HPV vaccination coverage in Japan after resumption of the government recommendation has remained modest [5]. Recent cohort-based national analyses report cumulative first-dose uptake of approximately 1–25% among the affected birth cohorts, with a theoretical upper bound of recovery until FY 2028 estimated at approximately 43% [8].

Several previous studies have suggested that parental attitudes—particularly those of mothers—play a critical role in HPV vaccine decision-making for daughters [9,10,11,12,13]. Other studies have also shown that, even among health care professionals, gaps in knowledge and awareness related to HPV vaccination persist [14,15]. However, many of these investigations were conducted prior to the reinstatement of active governmental recommendation, and evidence under the current policy environment remains limited. In the present study, we aimed to assess HPV vaccine acceptability and to identify factors associated with acceptance among staff at a university hospital, with the aim to provide insights relevant to improving HPV vaccination coverage in Japan.

## 2. Materials and Methods

### 2.1. Participants and Setting

We administered a cross-sectional questionnaire survey on HPV vaccination among staff employed at Yokohama City University Hospital between 12 February and 29 February 2024. The head count of all hospital departments identified a total of 2761 staff members. All staff were eligible to participate regardless of employment status (full-time, part-time, or contract-based), occupation (e.g., physician, nurse, radiologic technologist, clinical laboratory technologist, administrative staff), or sex. Individuals who completed the questionnaire and provided informed consent to participate in the study were included in the analysis. No exclusion criteria were applied.

### 2.2. Procedure

An explanation of the study, along with a quick response (QR) code linking to the online questionnaire, was distributed to each department either in person or via the hospital’s internal mail system. Participation was voluntary, and those who agreed provided their responses using an anonymous web-based form. A reminder notice was sent out using the same method during the survey period to encourage participation among all eligible staff members who had not yet completed the questionnaire.

### 2.3. Questionnaire

The questionnaire had four parts. The first part comprised 10 questions on basic knowledge about cervical cancer and HPV vaccination that were developed on the basis of our group’s earlier studies [16,17,18,19] and informed by previous studies [20,21]. Respondents were asked to answer the questions (shown in Table S1) with “correct,” “incorrect,” or “don’t know.” The second part included questions about respondents’ awareness: whether they had heard about the effectiveness of the HPV vaccine, whether they had heard about possible adverse events after vaccination, and whether they had heard about catch-up vaccination programs, as described in past studies [22]. The third part consisted of questions regarding the acceptability of HPV vaccination for respondents’ hypothetical daughters, presenting four hypothetical scenarios and inviting responses.

Scenario 1: “Suppose you have a 14-year-old daughter who has not received an HPV vaccine. The local government provides information about free routine HPV vaccination, which is available until the end of her first year of high school in Japan. Would you want your daughter to receive the HPV vaccine within this timeframe?”
Scenario 2: “Suppose you have a 20-year-old daughter who has not received an HPV vaccine. The local government offers free catch-up HPV vaccination, available until the end of March 2025. Would you want your daughter to receive the vaccine within this timeframe?”
Scenario 3: “Suppose you have a 28-year-old daughter who has not received an HPV vaccine. The HPV vaccination would cost approximately JPY 50,000 (USD 315, based on an exchange rate of JPY 155 per USD) for three doses (quadrivalent vaccine). Would you want your daughter to receive the vaccine?”
Scenario 4: “Suppose you have a 28-year-old daughter who has not received an HPV vaccine. The HPV vaccination would cost approximately JPY 100,000 (USD 630) for three doses (9-valent vaccine). Would you want your daughter to receive the vaccine?”

The scenarios were constructed based on the contingent valuation method [23,24]. Respondents chose one of three options for each scenario: “I would like her to be vaccinated,” “I would not like her to be vaccinated,” or “Prefer not to answer.” These scenarios reflect actual HPV vaccination programs in Japan. The last part included questions on demographic characteristics such as sex, age, profession, and presence of a daughter (i.e., whether the respondent had at least one daughter). Because this was a single-center survey, daughters’ ages were not directly collected to minimize the risk of participant identification. All questions were mandatory, and most items included response options such as “do not know,” “not specified,” or “prefer not to answer.”

### 2.4. Statistical Analysis

In this study, for questions about hypothetical situations, a respondent’s choice of “I would like her to be vaccinated” was considered to be a “yes” response and acceptance of a given scenario. However, answers of “I would not like her to be vaccinated” and “Prefer not to answer” were deemed to indicate no acceptance of the scenario. Factors potentially influencing the probability of “yes” or acceptability were analyzed for each scenario using binary logistic regression models, where 1 represents a “yes” response and 0 otherwise. Explanatory variables in the regression models were selected based on previous studies and their potential relevance to HPV vaccine acceptability and included respondents’ characteristics, knowledge levels about cervical cancer and HPV vaccination, and awareness about HPV vaccination (effectiveness, adverse events, and the catch-up program). The final models included sex, age, occupation, having a daughter, and knowledge test score, as well as awareness of vaccine effectiveness, adverse events, and the catch-up program. Sex and age were treated as background factors, whereas knowledge and awareness variables were conceptualized as modifiable factors. Occupation and having a daughter were also included as adjustment factors that may influence acceptability. These factors were categorized and expressed as categorical variables. Responses of “not specified” or “prefer not to answer” were coded as 0 within the predefined categories. The percentage of “yes” responses was defined as the acceptability rate (AR) of each scenario and was presented with 95% confidence intervals (CIs). The Mann–Whitney U test was used to compare the knowledge test scores between groups. We considered *p*-values of <0.05 to indicate statistical significance. IBM SPSS Statistics 29.0 (IBM Corp., Armonk, NY, USA) was used for the analyses.

### 2.5. Ethical Approval

This study was approved by the Institutional Review Board for Life Science and Medical Research Involving Human Subjects at Yokohama City University (Approval No. F231100034). Participants accessed the online questionnaire via a QR code, read the explanatory document, checked the “I agree” box, and then began completing the questionnaire. Respondents were allowed to select options such as “Do not know” or “Prefer not to answer.” Because the survey was anonymous, consent could not be withdrawn after the survey was submitted.

### 2.6. Use of Generative AI

A generative artificial intelligence (AI) tool (ChatGPT-5.2, OpenAI, San Francisco, CA, USA; https://chat.openai.com, accessed on 17 February 2026) was used to support the literature search, text drafting, translation from Japanese to English, and to provide general advice on statistical procedures (e.g., IBM SPSS operation and interpretation). All analyses were performed by the authors using IBM SPSS, and all AI-generated suggestions were reviewed and verified by the authors. Generative AI was not used for the actual data analysis, study design, data collection, or interpretation of the statistical analysis results.

## 3. Results

The questionnaire packets were distributed to 2761 staff members, 1153 of whom accessed the survey via Microsoft Forms (Microsoft Corporation, Redmond, WA, USA; https://forms.microsoft.com/, accessed on 17 February 2026). Among them, 21 declined to participate at the consent stage; thus, responses from the remaining 1132 participants were included in the final analysis (response rate: 41.0%).

### 3.1. Respondent Characteristics

Table 1 summarizes the characteristics of respondents. Among the 1132 respondents, 812 were women (71.7%), 304 were men (26.9%), and 16 (1.4%) did not report their sex. The age distribution was as follows: 225 (19.9%) were aged 20–29 years, 244 (21.6%) aged 30–39 years, 307 (27.1%) aged 40–49 years, 263 (23.2%) aged 50–59 years, and 93 (8.2%) were aged ≥ 60 years. No respondents were younger than 20 years of age. Occupations were categorized into four groups: 222 (19.6%) respondents were medical doctors (including physicians, dentists, and residents), 338 (29.9%) were registered nurses or midwives, 172 (15.2%) were other medical professionals (such as pharmacists and technicians), and 400 (35.3%) were non-medical staff, including clerical workers, kitchen assistants, logistics personnel, and custodial staff. A total of 394 respondents (34.8%) reported having a daughter, 718 (63.4%) did not have a daughter, and 20 (1.8%) did not respond to the question. Respondents’ characteristics expressed as categorical variables are presented in Table 1.

### 3.2. Demographics, Knowledge, and Awareness by Occupation

Table 2 presents the distributions of sex, age, and responses to knowledge-related or awareness-related questions on HPV vaccination and cervical cancer across occupational groups. Based on the number of correct answers to 10 questions on basic knowledge about cervical cancer and HPV vaccination, respondents were classified into three groups using the tertile method: those with a low score (0–5 points; *n* = 265), medium score (6–8 points; *n* = 420), and high score (9–10 points; *n* = 447). Results of the Mann–Whitney U-test showed that the knowledge test scores of medical professionals (doctors/dentist, registered nurses/midwives, and other medical professionals) were significantly higher than those of non-medical staff (median = 9 vs. 6, *p* < 0.001). There was no significant difference in knowledge test scores between women and men (median = 8 for both, *p* = 0.303).

### 3.3. Differences in Responses Across Four Hypothetical Scenarios

**Figure 1:**
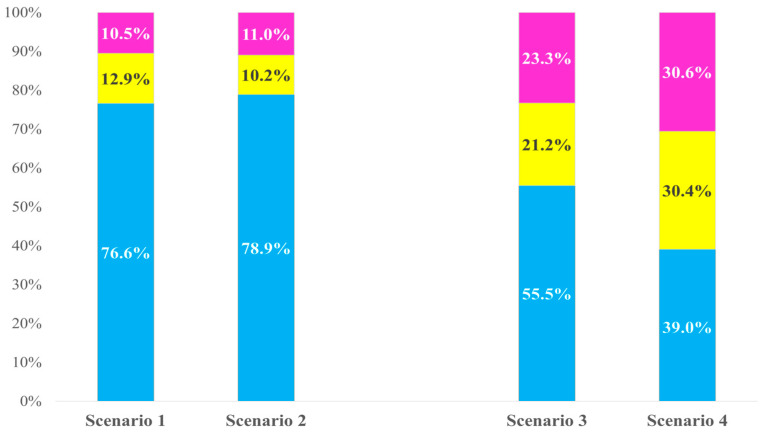
Differences in Responses Across Four Hypothetical Scenarios.

Figure 1 shows the percentage for each of the three response options (“I would like her to be vaccinated,” “I would not like her to be vaccinated,” and “Prefer not to answer”) in relation to HPV vaccination for a hypothetical daughter under the four scenarios. The proportion of respondents selecting “I would like her to be vaccinated” was 76.6% in Scenario 1 and 78.9% in Scenario 2, indicating similar levels of acceptability under the two publicly funded scenarios. The proportions declined substantially in the self-funded scenarios: 55.5% in Scenario 3 (quadrivalent, JPY 50,000 [USD 315]) and 39.1% in Scenario 4 (9-valent, JPY 100,000 [USD 630]). The acceptability was lower for Scenario 4 than that for Scenario 3.

Percentage of answers to questions on human papillomavirus (HPV) vaccination for a hypothetical daughter across four scenarios (*N* = 1132).

Blue: “I would like her to be vaccinated”; yellow: “I would not like her to be vaccinated”; pink: “Prefer not to answer.”

Scenario 1, routine vaccination; Scenario 2, catch-up vaccination; Scenario 3, quadrivalent vaccine, JPY 50,000 (USD 315); Scenario 4, 9-valent vaccine, JPY 100,000 (USD 630). 

Percentages may not total 100% due to rounding.

### 3.4. Factors Influencing Vaccine Acceptability

Results of the multivariable logistic regression analyses conducted for each scenario are presented in Table 3 and Table 4. There was no evidence of multicollinearity; a sample correlation coefficient between any pair of explanatory variables was less than 0.70 and greater than −0.70. Regression diagnostics are provided in the Supplementary Materials, including variance inflation factors for the explanatory variables (Table S2) and model fit indices (Hosmer–Lemeshow test, Cox and Snell and Nagelkerke pseudo R^2^ measures, and omnibus test) for all four scenarios (Table S3).

Scenarios 1 and 2 described publicly funded vaccination programs. Scenario 1 represents the routine vaccination program, whereas Scenario 2 represents the catch-up program. The analyses showed similar results for both scenarios (Table 3). Respondents aged 30 years and older were significantly less likely to accept these vaccination scenarios, as compared with those aged 20–29 years (*p* < 0.05). Compared with non-medical staff, medical professionals were more likely to accept these scenarios, with higher adjusted odds ratios among both medical doctors/dentists and other medical professionals (*p* < 0.05). Respondents who had a daughter were more likely to accept vaccination, although statistical significance was achieved only in Scenario 1 (*p* = 0.014). Compared with respondents in the low-score group for basic knowledge questions, those in the high-score groups were more likely to accept the scenario (*p* < 0.001). Those who had heard about effectiveness of the vaccine or the catch-up program were more likely to accept vaccination (*p* < 0.05). By contrast, hearing about adverse events had a negative impact on vaccine acceptance (*p* < 0.001).

Scenarios 3 and 4 described self-funded vaccination programs. In Scenario 3, JPY 50,000 (USD 315) was charged for the quadrivalent vaccine, and in Scenario 4, JPY 100,000 (USD 630) was charged for the 9-valent vaccine. The analyses showed similar results for these two scenarios (Table 4). Women were significantly less likely than men to accept these vaccination scenarios (*p* < 0.001). There were no statistically significant differences in acceptability across age groups. Having a daughter did not show a significant difference in vaccine acceptability. Compared with non-medical staff, all medical professionals were more likely to accept these scenarios, although statistical significance was not achieved for registered nurses in Scenario 3. Compared with the group with low scores for basic knowledge questions, respondents in the high-score group were more likely to accept vaccination (*p* < 0.05). Respondents who had heard about effectiveness of the vaccine and the catch-up program were also more likely to accept the catch-up scenario (Scenario 3, *p* < 0.05). Hearing about adverse events did not have a significant impact on vaccine acceptance.

### 3.5. Vaccine Acceptability and Respondent Characteristics

**Figure 2:**
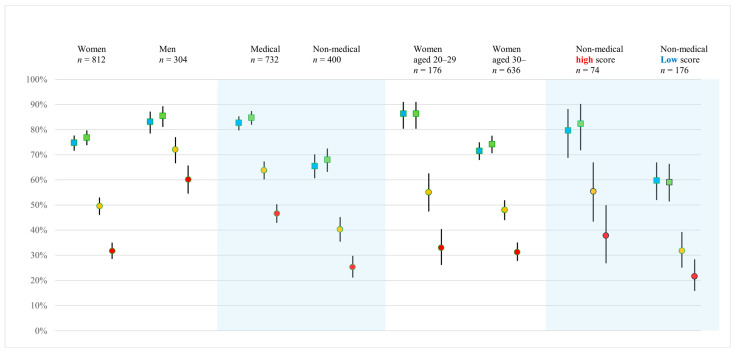
Respondents’ Characteristics and Vaccine Acceptability.

Figure 2 shows the differences in vaccine acceptability according to respondents’ characteristics in each scenario. Acceptance rates of the scenarios are displayed with 95% CIs. Only representative and distinctive characteristic patterns are displayed. Overall, the acceptance rates for self-funded vaccination programs (Scenarios 3 and 4) were lower than those for publicly funded vaccination programs (Scenarios 1 and 2). More than 70% of female respondents reported acceptance for Scenarios 1 and 2 (AR: 74.8%, 95% CI: 71.6–77.7% and AR: 76.8%, 95% CI: 74.3–80.2%, respectively). Approximately 80% of male respondents reported acceptance for Scenarios 1 and 2 (AR: 83.2%, 95% CI: 78.5–87.2% and AR: 85.5%, 95% CI: 81.1–89.3%, respectively). Acceptance rates were substantially decreased in Scenarios 3 and 4, especially among women (AR: 49.5%, 95% CI: 46.0–53.0% and AR: 31.7%, 95% CI: 28.5–38.5%, respectively). When comparing occupational groups, medical professionals had greater vaccine acceptance than non-medical staff in all scenarios. Among non-medical staff, fewer than half reported acceptance for the self-funded vaccination programs in Scenarios 3 and 4. Regarding vaccine acceptance among women, those aged 20–29 years had higher acceptance rates for publicly funded vaccination programs (Scenarios 1 and 2) than women aged 30 years and older. However, in scenarios involving self-funded vaccination programs (Scenarios 3 and 4), acceptance rates among younger women declined to levels comparable to those of older age groups. Acceptability among non-medical staff is shown as a comparison between those with high and low scores on basic knowledge questions. The high-score group tended to have high acceptance rates across all scenarios in comparison with the low-score group. No substantial differences in vaccine acceptability for either sex were observed according to whether respondents had a daughter (shown in Figure S1).

Acceptability of HPV vaccination for a hypothetical daughter across four scenarios, shown by representative respondent characteristics. Error bars indicate 95% confidence intervals.

Blue square: acceptability rate in Scenario 1 (publicly funded vaccination), green square: acceptability rate in Scenario 2 (publicly funded vaccination), orange circle: acceptability rate in Scenario 3 (self-funded vaccination), red circle: acceptability rate in Scenario 4 (self-funded vaccination).

### 3.6. Knowledge, Awareness, and Vaccine Acceptability

**Figure 3:**
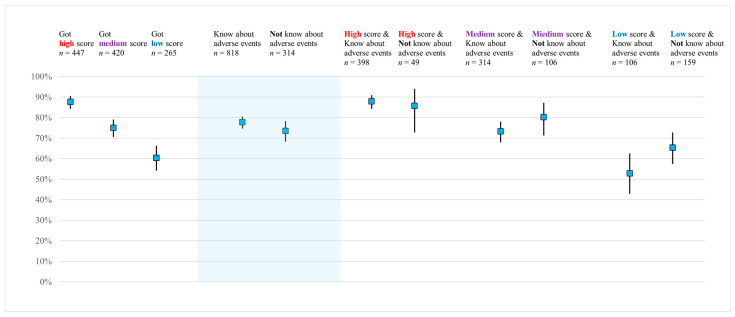
Knowledge, Awareness, and Vaccine Acceptability.

Figure 3 describes how basic knowledge and awareness of adverse events influenced vaccine acceptability in a routine vaccination program (Scenario 1). Respondents in the high-score group for basic knowledge questions demonstrated significantly higher acceptance rates, with a clear gradient observed across the high-, medium-, and low-score groups. With respect to awareness of adverse events, those who had heard about adverse events showed slightly higher acceptance rates than those who had not. When stratified by both knowledge score and awareness of adverse events, the acceptance rates in the high-score group remained consistently high regardless of awareness about adverse events. In the medium- and low-score groups, respondents who were aware of adverse events tended to show lower acceptance rates than those who were unaware of adverse events.

Acceptability of HPV vaccination in Scenario 1 (routine vaccination), shown by knowledge levels and awareness about adverse events. Blue square: acceptability rate. Error bars indicate 95% confidence intervals.

## 4. Discussion

In this study, the acceptability levels of HPV vaccination for a hypothetical daughter were substantially higher in publicly funded scenarios than those in self-funded scenarios. Although direct comparisons are limited owing to differences in study design, acceptability in the routine vaccination scenario appeared higher than that reported in a recent nationwide survey in Japan [25]. Our study population included staff at a university hospital and therefore differed from the general population with respect to several background characteristics, including sex and occupational distribution, with a high proportion of women and medical professionals; these differences were accounted for in the multivariable analyses. Multivariable analyses identified age, sex, occupation, knowledge level, and awareness about HPV vaccination as factors independently associated with acceptability, with partial differences in associated factors between publicly funded and self-funded scenarios. The implications of these findings are discussed below.

Regarding age, in the publicly funded scenarios, respondents aged 30 years and above had significantly lower adjusted odds ratios of acceptance, as compared with those aged 20–29 years (Table 3). A similar tendency was observed when comparing acceptance rates according to age among women (Figure 2). Individuals over 30 years old are currently parents or will become parents in the near future. Previous studies have frequently shown that parental attitudes strongly influence vaccination behaviors for daughters [9,10,11,12,13]. Therefore, the lower acceptability observed in this age group suggests the importance of engaging parent-aged populations in efforts to improve vaccination coverage among eligible female adolescents.

With respect to sex, acceptability among women tended to be lower than that among men and was significantly lower in the self-funded scenarios (Table 4). Figure 2 shows that in self-funded scenarios, acceptability was more affected for women than for men. In Scenario 3 (quadrivalent, JPY 50,000 [USD 315]), the acceptability rate among women was approximately 50%; this decreased further to approximately 30% in Scenario 4 (9-valent, JPY 100,000 [USD 630]), despite the broader protection offered by the 9-valent vaccine. This pattern suggests that financial burden plays an important role, particularly among women. Cost has been reported as a potentially strong barrier to HPV vaccination uptake [10,11,12], and our findings further suggest that this effect is especially pronounced in the context of decision-making among mothers. However, because the four scenarios differed in terms of the daughter’s age and type of vaccine, the independent impact of cost cannot be assessed. Historically, HPV vaccination awareness campaigns have primarily targeted women [26,27]. Numerous studies have reported that women generally possess greater HPV-related knowledge [28,29,30] and are more proactive in making vaccination-related decisions for their daughters [12,31]. In the present study, however, no sex differences were observed in basic knowledge scores, indicating a pattern distinct from that of the general population. The greater acceptability among men in this study is consistent with findings from a Japanese study by Suzuki et al. [18], who reported that although men had lower knowledge levels, they were more supportive of vaccinating their daughters after adjusting for knowledge and other factors. Furthermore, two randomized controlled trials by the same researchers showed that provision of brief information and a web-based story involving a survivor of cervical cancer significantly increased men’s vaccination intentions [19,32]. Taken together, these findings imply that although women generally exhibit greater knowledge and willingness, men may have increased acceptance once provided with appropriate information. Additionally, in Japan, the indication for the 9-valent vaccine was expanded to males from August 2025, and an increasing number of municipalities are introducing subsidies for male HPV vaccination. These developments may contribute to improved vaccination rates among both women and men.

Knowledge level emerged as a factor associated with acceptability in all scenarios. A tendency was observed in that the higher the knowledge score, the greater the acceptability of vaccination (Figure 3). Medical professionals had higher knowledge scores than non-medical staff (Figure 2), suggesting a relationship between occupation and knowledge. However, multivariable analyses showed that occupation and knowledge each had a significant effect. Even among non-medical staff, respondents with greater knowledge scores showed greater acceptability (Figure 2). This is consistent with many previous reports showing a positive association between HPV-related knowledge and vaccination intention [10,11,12,33].

Regarding awareness about HPV vaccination, awareness about effectiveness and catch-up vaccination positively influenced acceptability, whereas awareness of adverse events had a negative influence on acceptability. In Figure 3, acceptance rates for HPV vaccination in Scenario 1 (routine vaccination) are displayed according to knowledge levels and awareness of adverse events. Respondents who were aware of adverse events showed slightly greater acceptability than other respondents, indicating a direction that differed from the negative association found in multivariable analysis. To clarify this discrepancy, acceptance rates were compared according to subgroups of knowledge level and adverse event awareness, revealing a pattern similar to Simpson’s paradox. Contrary to the overall pattern, respondents with insufficient knowledge (medium- and low-score groups) tended to have lower acceptability. By contrast, respondents with sufficient knowledge (high-score group) showed high acceptability, regardless of whether they were aware of adverse events. This observation suggests that the meaning of “having heard about adverse events” may vary depending on underlying knowledge levels, and that awareness of adverse events does not necessarily reduce acceptability when respondents possess sufficient knowledge. Previous studies have reported that vague safety concerns and exaggerated or incorrect perceptions about adverse events are associated with lower vaccination intentions [10,11,12,34], but scientifically balanced information can alleviate such concerns and enhance acceptance [35,36]. Collectively, these findings suggest that the quality of underlying knowledge and the credibility of information sources—rather than simply whether one has heard about possible adverse events—may be key determinants of vaccination intention.

In this study, we applied the contingent valuation method, which is commonly used to assess willingness-to-pay in cost–benefit analyses. This method is a practical approach to measuring willingness-to-pay that considers individual and societal preferences [37,38]. However, we used contingent valuation to measure factors influencing program acceptability rather than measuring willingness-to-pay. Although hypothetical questions were asked, only the situations were hypothetical; the programs themselves actually exist. The dichotomous-choice or “take-it-or-leave-it” approach is one of the main contingent valuation methods [39,40]. We applied a take-it-or-leave-it approach to assess HPV vaccination acceptability, in which participants were presented with a hypothetical scenario and asked to give a dichotomous “yes” or “no” response, but they were also given a “no answer” option. A disadvantage of the take-it-or-leave-it approach is the extent to which hypothetical choices represent real choices. Some studies suggest that hypothetical choices outweigh actual choices, a phenomenon known as hypothetical bias [41]. There are several ways to address hypothetical bias. For example, overestimation can be corrected by sorting “definitely” affirmative responses, as identified in a simple follow-up question [42]. The present survey allowed respondents to select “Prefer not to answer,” which may reduce the discrepancy between a hypothetical “yes” and a real “yes.”

This study has several limitations. First, this research was conducted at a single university hospital and involved a specific population whose demographic composition differed from that of the general population. In addition, the response rate was 41%, raising the possibility of self-selection bias, as individuals with greater interest in vaccination may have been more likely to participate. Because this was an in-hospital survey, social desirability bias may also have influenced the responses. Second, the categorization of scores may generally be perceived as arbitrary. In this study, we attempted to objectively categorize the number of correct answers to 10 basic knowledge items using the tertile method. Third, we assessed vaccination intentions using hypothetical scenarios rather than actual vaccination behavior. A survey of parents whose daughters actually fit into each of the four scenarios might provide more precise estimates of real-world decision-making. Fourth, the questionnaire used in this study was not formally validated. Although household income was not directly assessed, occupation was included as a proxy indicator of socioeconomic status. Future studies can address these limitations by expanding the study population to more representative general populations or by focusing on individuals who are directly eligible for vaccination and their parents. Despite these limitations, the study’s strengths include its large, diverse sample of hospital staff and its multifaceted examination of HPV vaccination acceptability.

Based on our findings, improving HPV vaccination coverage in Japan—particularly among adolescents who are eligible for routine vaccination—will require approaches tailored to population characteristics. To achieve this, parent-aged adults constitute a critical target group, and the types of effective intervention may differ according to sex. For men, it is important to first provide sufficient foundational information about HPV vaccination, followed by encouraging active participation in vaccination decision-making in their role as fathers. For women, clearly communicating the time-limited opportunity for publicly funded vaccination may promote timely behavioral change. Across all populations, the provision of scientifically balanced information is essential. In addition to explaining vaccine effectiveness, offering appropriate explanations of the scientific context behind past reports of adverse events may help to prevent misunderstanding, deepen comprehension, and ultimately enhance acceptability.

## 5. Conclusions

In this study, acceptability of HPV vaccination for a hypothetical daughter was high in publicly funded scenarios but substantially lower in self-funded scenarios. Based on the overall results, tailored approaches may be necessary to improve HPV vaccination coverage, with knowledge-enhancing interventions being potentially effective for men and strategies to address cost-related barriers being especially important for women. Providing scientifically balanced information, including evidence regarding vaccine effectiveness and clear explanations of the scientific context behind previous reports of adverse events, may further help to improve HPV vaccine acceptability across all population groups.

## Figures and Tables

**Table 1:** Demographic characteristics, occupational groups, and presence of daughters among respondents (*N* = 1132).

	*n*	%	Categorical Variable
Sex			
Female	812	71.7%	1
Male	304	26.9%	0
Not specified	16	1.4%	0
Age (years)			
60 or older	93	8.2%	5
50 to 59	263	23.2%	4
40 to 49	307	27.1%	3
30 to 39	244	21.6%	2
20 to 29	225	19.9%	1
Profession			
Medical doctor, Dentist	222	19.6%	4
Registered nurse, Midwife	338	29.9%	3
Other medical professional	172	15.2%	2
Pharmacist	31	2.7%	2
Medical Laboratory Scientist, Radiologic Technologist, Clinical Engineer	96	8.5%	2
Physical Therapist, Occupational Therapist, Speech-Language Pathologist, Orthoptist,	22	1.9%	2
Dietitian	11	1.0%	2
Social Worker, Psychiatric Social Worker, Psychologist	12	1.1%	2
Other occupations (non-medical staff)	400	35.3%	1
Administrative staff, medical clerk, reception staff	234	20.7%	1
Nursing assistant	34	3.0%	1
Linen services, supply/processing/distribution services, logistics staff	32	2.8%	1
Cleaning staff	16	1.4%	1
Cook, food preparation assistant, meal delivery staff	9	0.8%	1
Other	75	6.6%	1
Presence of a daughter			
Yes	394	34.8%	1
No	718	63.4%	0
No answer	20	1.8%	0

**Table 2:** Demographic characteristics, knowledge levels, and awareness about human papillomavirus (HPV) vaccination across occupational groups.

	Medical Doctor /Dentist (*n* = 222)		Registered Nurse /Midwife (*n* = 338)		Other Medical Professionals (*n* = 172)		Other Occupations (Non-Medical Staff) (*n* = 400)	
Sex								
Female	86	38.7%	316	93.5%	97	56.4%	313	78.3%
Male	135	60.8%	17	5.0%	72	41.9%	80	20.0%
Not specified	1	0.5%	5	1.5%	3	1.7%	7	1.8%
Age (years)								
60 or older	5	2.3%	13	3.8%	12	7.0%	63	15.8%
50 to 59	19	8.6%	72	21.3%	39	22.7%	133	33.3%
40 to 49	78	35.1%	82	24.3%	37	21.5%	110	27.5%
30 to 39	88	39.6%	56	16.6%	46	26.7%	54	13.5%
20 to 29	32	14.4%	115	34.0%	38	22.1%	40	10.0%
Knowledge test scores								
High score (9–10 points)	159	71.6%	136	40.2%	78	45.3%	74	18.5%
Medium score (6–8 points)	54	24.3%	148	43.8%	68	39.5%	150	37.5%
Low score (0–5 points)	9	4.1%	54	16.0%	26	15.1%	176	44.0%
Have you heard about the effectiveness of the HPV vaccine?								
Yes	188	84.7%	223	66.0%	117	68.0%	195	48.8%
No	33	14.9%	102	30.2%	53	30.8%	189	47.3%
No answer	1	0.5%	13	3.8%	2	1.2%	16	4.0%
Have you heard about adverse events following HPV vaccination?								
Yes	188	84.7%	257	76.0%	127	73.8%	246	61.5%
No	32	14.4%	73	21.6%	43	25.0%	147	36.8%
No answer	2	0.9%	8	2.4%	2	1.2%	7	1.8%
Are you aware of the catch-up HPV vaccination program?								
Yes	134	60.4%	155	45.9%	69	40.1%	155	38.8%
No	87	39.2%	179	53.0%	98	57.0%	237	59.3%
No answer	1	0.5%	4	1.2%	5	2.9%	8	2.0%

Note: Medical professionals include medical doctors/dentists, registered nurses/midwives, and other licensed medical professionals. Non-medical staff include administrative, clerical, support, logistics personnel, and others. Knowledge levels were categorized based on tertiles.

**Table 3:** Multivariable logistic regression models for Scenario 1 (routine vaccination) and Scenario 2 (catch-up vaccination).

	Scenario 1			Scenario 2		
Variable	Adjusted OR	(95% CI)	*p*-Value	Adjusted OR	(95% CI)	*p*-Value
Female sex	0.879	(0.594–1.299)	0.517	0.767	(0.509–1.158)	0.207
Age (years)						
60 or older	0.397	(0.204–0.774)	0.007	0.464	(0.235–0.917)	0.027
50–59	0.325	(0.192–0.550)	<0.001	0.421	(0.245–0.724)	0.002
40–49	0.439	(0.262–0.736)	0.002	0.561	(0.328–0.958)	0.034
30–39	0.387	(0.227–0.662)	<0.001	0.433	(0.249–0.751)	0.003
20–29	1			1		
Profession						
Medical doctor/Dentist	2.554	(1.408–4.635)	0.002	2.538	(1.337–4.818)	0.004
Registered nurse/Midwife	1.160	(0.797–1.689)	0.438	1.151	(0.780–1.697)	0.479
Other medical professionals	2.342	(1.385–3.960)	0.001	2.390	(1.368–4.178)	0.002
Other occupations (non-medical staff)	1			1		
Presence of a daughter	1.527	(1.090–2.140)	0.014	1.271	(0.896–1.803)	0.179
Knowledge test scores						
High score (9–10 points)	2.375	(1.451–3.889)	<0.001	3.192	(1.909–5.339)	<0.001
Medium score (6–8 points)	1.403	(0.950–2.072)	0.089	1.864	(1.252–2.775)	0.002
Low score (0–5 points)	1			1		
Have you heard about the effectiveness of HPV vaccines? “Answer = yes”	2.387	(1.659–3.434)	<0.001	2.200	(1.510–3.205)	<0.001
Have you heard about adverse events following HPV vaccination? “Answer = yes”	0.454	(0.304–0.678)	<0.001	0.455	(0.301–0.687)	<0.001
Are you aware of the catch-up HPV vaccination program? “Answer = yes”	1.468	(1.022–2.107)	0.038	1.581	(1.081–2.313)	0.018

Reference categories: 20–29 years (age), non-medical staff (profession), low knowledge score (0–5 points). Abbreviations: OR, odds ratio; CI, confidence interval.

**Table 4:** Multivariable logistic regression models for Scenario 3 (quadrivalent vaccine, JPY 50,000 [USD 315]) and Scenario 4 (9-valent vaccine, JPY 100,000 [USD 630]).

	Scenario 3			Scenario 4		
Variable	Adjusted OR	(95% CI)	*p*-Value	Adjusted OR	(95% CI)	*p*-Value
Female sex	0.461	(0.331–0.642)	<0.001	0.425	(0.308–0.587)	<0.001
Age (years)						
60 or older	0.978	(0.558–1.716)	0.939	1.005	(0.658–1.537)	0.980
50–59	0.819	(0.538–1.246)	0.351	0.958	(0.620–1.480)	0.847
40–49	0.807	(0.540–1.208)	0.298	1.042	(0.690–1.571)	0.846
30–39	0.853	(0.560–1.300)	0.460	1.005	(0.658–1.537)	0.980
20–29	1			1		
Profession						
Medical doctor/Dentist	2.548	(1.609–4.034)	<0.001	3.270	(2.095–5.106)	<0.001
Registered nurse/Midwife	1.406	(1.007–1.963)	0.046	1.287	(0.895–1.849)	0.173
Other medical professionals	1.800	(1.193–2.717)	0.005	1.709	(1.127–2.594)	0.012
Other occupations (non-medical staff)	1			1		
Presence of a daughter	0.894	(0.668–1.196)	0.450	0.906	(0.671–1.224)	0.521
Knowledge test scores						
High score (9–10 points)	2.595	(1.689–3.987)	<0.001	2.045	(1.296–3.228)	0.002
Medium score (6–8 points)	1.771	(1.226–2.559)	0.002	1.339	(0.892–2.011)	0.159
Low score (0–5 points)	1			1		
Have you heard about the effectiveness of HPV vaccines? “Answer = yes”	1.533	(1.109–2.120)	0.010	1.365	(0.968–1.925)	0.076
Have you heard about adverse events following HPV vaccination? “Answer = yes”	0.709	(0.501–1.005)	0.053	0.872	(0.607–1.253)	0.459
Are you aware of the catch-up HPV vaccination program? “Answer = yes”	1.359	(1.007–1.833)	0.045	1.384	(1.016–1.884)	0.039

Reference categories: 20–29 years (age), non-medical staff (profession), low knowledge score (0–5 points). Abbreviations: OR, odds ratio; CI, confidence interval.

## Data Availability

The data presented in this study are available on request from the corresponding author. The data are not publicly available owing to ethical and institutional restrictions, as they involve internal survey responses from staff of Yokohama City University Hospital. Anonymized data may be provided upon reasonable request and with permission from the institution.

## Supplementary Materials

The following supporting information can be downloaded at: https://www.mdpi.com/article/10.3390/vaccines14030218/s1, Figure S1. Respondents’ Sex, Presence of a Daughter, and Vaccine Acceptability; Table S1. Basic knowledge questions; Table S2. Variance inflation factors (VIFs) for explanatory variables. Table S3. Model fit indices for multivariable logistic regression models in the four scenarios.

## Abbreviations

The following abbreviations are used in this manuscript:

HPV	human papillomavirus
WHO	World Health Organization
QR code	quick response code
AR	acceptability rate
CI	confidence interval
OR	odds ratio
VIF	Variance inflation factor
