# Supplementary material for: Acceptability of HPV Vaccination for Daughters: A University Hospital-Wide Questionnaire Survey
Source: Vaccines (Basel). 2026 Feb 27;14(3):218. doi: 10.3390/vaccines14030218 (PMC13030174; doi:10.3390/vaccines14030218)
Supplement: Supplementary file 1 [file vaccines-14-00218-s001.zip › vaccines-4116026-supplementary-2.27/Figure S1 (vaccines-4116026).pdf]

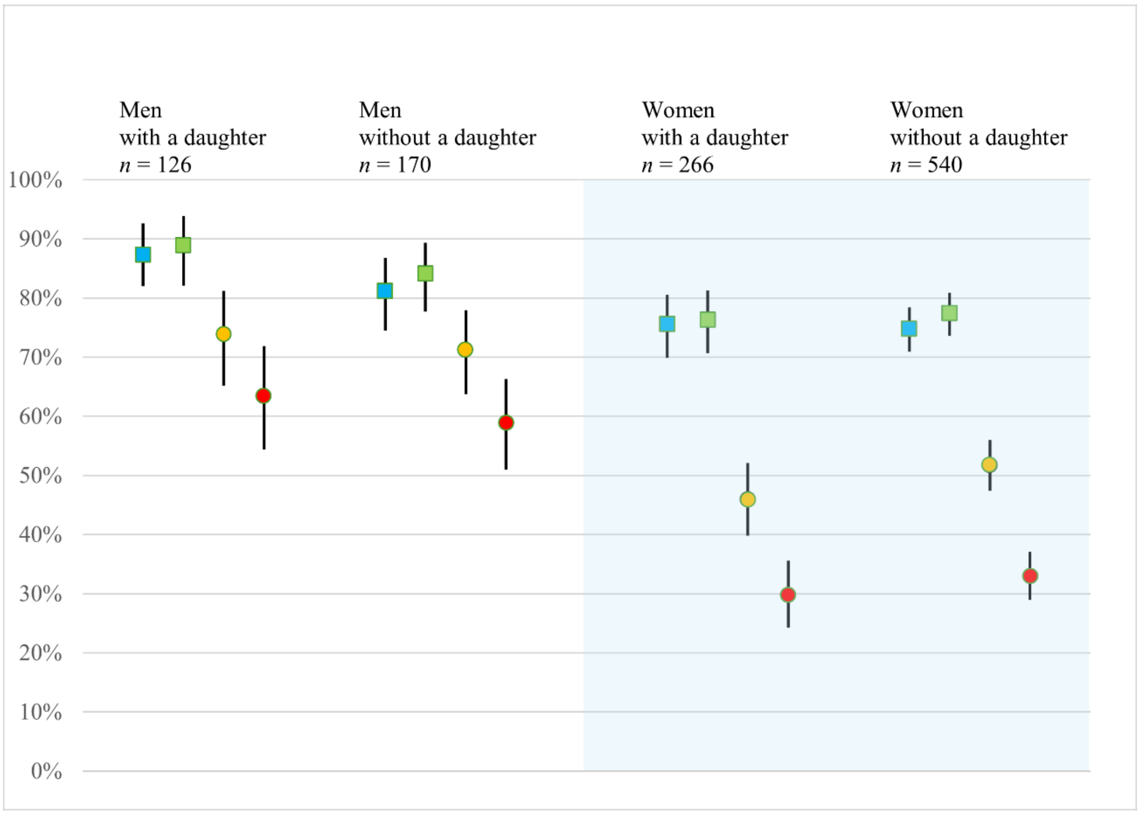

Figure S1. Respondents' Sex, Presence of a Daughter, and Vaccine Acceptability.

Acceptability of HPV vaccination for a hypothetical daughter across four scenarios, shown according to respondents' sex and presence of a daughter. Error bars indicate 95% confidence intervals. Blue square: acceptability rate in Scenario 1 (publicly funded vaccination), green square: acceptability rate in Scenario 2 (publicly funded vaccination), orange circle: acceptability rate in Scenario 3 (self-funded vaccination), red circle: acceptability rate in Scenario 4 (self-funded vaccination).
