# Supplementary material for: Acceptability of HPV Vaccination for Daughters: A University Hospital-Wide Questionnaire Survey
Source: Vaccines (Basel). 2026 Feb 27;14(3):218. doi: 10.3390/vaccines14030218 (PMC13030174; doi:10.3390/vaccines14030218)
Supplement: Supplementary file 1 [file vaccines-14-00218-s001.zip › vaccines-4116026-supplementary-2.27/Table S1 (vaccines-4116026).pdf]

Table S1. Basic knowledge questions

	Basic knowledge questions	
Q1	The incidence of cervical cancer is increasing among women in their 20s to 40s.	True
Q2	Human papillomavirus (HPV) infection is associated with the development of cervical cancer.	True
Q3	Women with a past history of cervical cancer can never become pregnant.	False
Q4	Sexual experience is associated with the risk of HPV infection.	True
Q5	Cervical cancer is the only cancer caused by HPV infection.	False
Q6	The HPV vaccine prevents infection with all types of HPV.	False
Q7	The HPV vaccine is more effective when administered before the onset of sexual activity.	True
Q8	In Japan, a catch-up vaccination program for HPV is available. (Note: The catch-up program provides opportunities to be vaccinated for individuals who missed routine HPV vaccination.)	True
Q9	Receiving the HPV vaccine completely eliminates the risk of developing cervical cancer.	False
Q10	Even if a woman has had sexual intercourse, young women do not need cervical cancer screening.	False
