# Supplementary material for: Acceptability of HPV Vaccination for Daughters: A University Hospital-Wide Questionnaire Survey
Source: Vaccines (Basel). 2026 Feb 27;14(3):218. doi: 10.3390/vaccines14030218 (PMC13030174; doi:10.3390/vaccines14030218)
Supplement: Supplementary file 1 [file vaccines-14-00218-s001.zip › vaccines-4116026-supplementary-2.27/Table S2 (vaccines-4116026).pdf]

Table S2. Variance inflation factors (VIFs) for explanatory variables.

Variable	VIF
Female sex	1.068
Age (years)	1.268
Profession	1.391
Presence of a daughter	1.168
Knowledge test scores	1.711
Have you heard about the effectiveness of HPV vaccines? "Answer = yes"	1.541
Have you heard about adverse events following HPV vaccination? "Answer = yes"	1.460
Are you aware of the catch-up HPV vaccination program? "Answer = yes"	1.357

VIF values were calculated using linear regression models with the explanatory variables included in the logistic regression models.
