# Supplementary material for: Acceptability of HPV Vaccination for Daughters: A University Hospital-Wide Questionnaire Survey
Source: Vaccines (Basel). 2026 Feb 27;14(3):218. doi: 10.3390/vaccines14030218 (PMC13030174; doi:10.3390/vaccines14030218)
Supplement: Supplementary file 1 [file vaccines-14-00218-s001.zip › vaccines-4116026-supplementary-2.27/Table S3 (vaccines-4116026).pdf]

Table S3. Model fit indices for multivariable logistic regression models in the four scenarios.

	Hosmer–Lemeshow test			Cox and Snell pseudo R^2	Nagelkerke pseudo R^2	Omnibus test		
	χ^2	df	p-value			χ^2	df	p-value
Scenario 1	9.771	8	0.281	0.129	0.195	156.901	14	< 0.001
Scenario 2	7.792	8	0.454	0.132	0.206	160.600	14	< 0.001
Scenario 3	15.000	8	0.059	0.139	0.186	169.117	14	< 0.001
Scenario4	4.952	8	0.763	0.150	0.204	184.319	14	< 0.001

Hosmer–Lemeshow test results, Cox and Snell and Nagelkerke pseudo R^2 measures, and omnibus test statistics are shown for each scenario.
